# Utility Rate Equations of Group Population Dynamics in Biological and Social Systems

**DOI:** 10.1371/journal.pone.0083225

**Published:** 2013-12-30

**Authors:** Vyacheslav I. Yukalov, Elizaveta P. Yukalova, Didier Sornette

**Affiliations:** 1 Department of Management, Technology and Economics, ETH Zürich, Swiss Federal Institute of Technology, Zürich, Switzerland; 2 Bogolubov Laboratory of Theoretical Physics, Joint Institute for Nuclear Research, Dubna, Russia; 3 Laboratory of Information Technologies, Joint Institute for Nuclear Research, Dubna, Russia; 4 Swiss Finance Institute, c/o University of Geneva, Geneva, Switzerland; University of Zaragoza, Spain

## Abstract

We present a novel system of equations to describe the evolution of self-organized structured societies (biological or human) composed of several trait groups. The suggested approach is based on the combination of ideas employed in the theory of biological populations, system theory, and utility theory. The evolution equations are defined as utility rate equations, whose parameters are characterized by the utility of each group with respect to the society as a whole and by the mutual utilities of groups with respect to each other. We analyze in detail the cases of two groups (cooperators and defectors) and of three groups (cooperators, defectors, and regulators) and find that, in a self-organized society, neither defectors nor regulators can overpass the maximal fractions of about 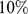 each. This is in agreement with the data for bee and ant colonies. The classification of societies by their distance from equilibrium is proposed. We apply the formalism to rank the countries according to the introduced metric quantifying their relative stability, which depends on the cost of defectors and regulators as well as their respective population fractions. We find a remarkable concordance with more standard economic ranking based, for instance, on GDP per capita.

## Introduction

Biological and human social systems have many common features allowing considering them on common grounds [1]. Actually, biological systems are nothing but particular kinds of social systems. And vice versa, human societies are particular types of biological systems. Therefore, the term social system can be applied to both examples of societies.

By definition, a biological or social system is an ensemble of entities characterized by their structure controlling enduring and relatively stable patterns of relationship between different groups of the society [2]–[6]. A social system is structured into several groups defined through their actions in mutual relations. The characteristics of these relations can change in the long run but, over limited time spans, they can be treated as fixed.

Any social system exists in an environment that certainly influences its properties. However, the definition of a social system presupposes that the influence of the environment does not preclude to identify the system as an entity, with its properties that are more or less specified for a sufficiently long time during which the system is well defined as such. That is, the notion of a social system implies that it can be defined as a self-organized entity, with given characteristics, which lives a sufficiently long time in order to be specified as a particular system. The system lifetime is much longer that the typical interaction time of its constituents, though it can be limited from above by the time scales at which the systems characteristics essentially vary. In that sense, the system lifetime is intermediate, being between the local variation time of its components and the time of the global change of its properties.

The structure and behavior of different societies, though being specific to each of them, nevertheless do possess general features that can be classified using mathematical methods, such that the variety of behaviors can be mapped onto differences in the system parameters and initial conditions. The concept, that social systems exhibit general reproducible behaviors and properties, has been emphasized in system theory [1], [7]–[13], which abstracts and considers a system as a connected set of interacting parts. The main goal of system theory is to study general principles of how systems function that can be applied to all types of systems from a given class.

Bertalanffy [1], who coined the term *system theory*, emphasized that biological systems have much in common with human social systems, as well as with separate biological organisms that can be treated as biological societies of different organs and cells. Dynamical models of biological organisms are also senseful at intermediate time scales that are longer than the characteristic functional times of the considered organism constituents, but shorter than the whole organism lifetimes [14], [15]. This is because during the whole organism lifetime, the parameters of the system can essentially vary, reflecting the aging processes. In the same way, the dynamical models of social systems are appropriate for describing intermediate time scales, during which the system parameters do not essentially change. Generally, the model structure can even be preserved for rather long times, of the order of the society lifetime, provided that the society parameters are slowly varying, so that it is possible to separate the system dynamics at different time scales, resorting to the scale separation approach [16], [17].

The classification, analysis, understanding and prediction of the evolution of biological societies, including human societies, is an old problem that has been treated in a voluminous literature starting from Darwin [18], [19]. Societies are usually structured into groups representing particular traits or strategies, so that such groups are termed *trait groups*, or *strategy groups*. Each group consists of agents with a specific trait and/or strategy. Typical group representatives are *collaborators* and *defectors*. The former collaborate with each other, contributing to the benefits of the whole society, motivated by self-interest and/or altruistic behavior. The latter, sometimes called free riders, do not contribute to the society but, on the contrary, exploit it, benefiting from it without enduring costs or making sacrifices.

Biological explanations of cooperation are based on kin altruism, reciprocal altruism, reputation or signaling, and mutualism, all of which apply to human as well as nonhuman species [20]. In humans, altruism can be enforced by internal norms [21]–[24]. According to the standard description, in a society, consisting of just two groups, cooperators and defectors, the latter always outcompete altruists [25]. The cooperation can be stabilized by the punishment of defectors [26]–[28]. There are, however, examples [29] when punishment decreases cooperation. While costly punishment, termed altruistic, may facilitate cooperation, at the same time, it can be interpreted as not so altruistic but rather selfish, because the original motivation behind punishment could be to retrieve deserved payoffs from their own contributions on the long run, which is a selfish incentive [30]–[32]. Punishment can also act indirectly, since the defectors, outcompeting cooperators inside a society, weaken the competitive ability of the society as a whole, as is considered in multilevel selection theory [25], [33]–[36].

The punishment of defectors can be realized directly by other members of the society. Since the ability of punishing can also be treated as a specific trait or strategy, it can be associated with a specific group of *punishers*. This is especially evident in complex societies, where punishers really form separate groups. For instance, in human societies, punishers are represented by police, law-enforcing services, and the army, which form what can be called the punisher group. The consideration of such trait groups does not forbid for the manifolds of cooperators and punishers to intersect [37], [38].

Sometimes, one considers one more specific trait group whose members are alienated from the society. The agents of such alienated groups, depending on their particular features, are termed by different names, as strangers [39], loners [40], outsiders [38], and by other similar terms. These *outsiders* do not contribute to the society, but exploit cooperators and are prosecuted by punishers. Their difference from defectors is that they enter the society from outside. There are plenty of examples of such outsiders in biological systems, where they are associated with pathogens or parasites [41].

The evolution of groups is usually studied within evolutionary game theory [42]–[45], whose continuous representation is given by the replicator equation [46]–[48]

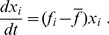
(1)


Here 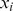 is the population fraction of group 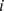 and 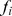 is the group fitness, and 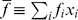 is the average fitness estimated over all groups. The group fractions are defined on the simplex. The trait-group fitness is often represented by the quadratic Lande-Arnold form widely used in describing the evolution of biological species, with the coefficients defined by calibration [49]–[51]. The most interesting information that can be extracted from this dynamics is the existence and properties of evolutionary stable strategies related to the stable stationary states of the dynamical system.

As is evident from the structure of the replicator equation (1), fitness is a quantity inversely proportional to time. This is concretized in the definition of fitness as being the product of viability and fecundity rate [52]–[55]. However, for short, one uses the term fitness, but not fitness rate. Similarly, below we shall use the term utility, but not utility rate. This will not be important for the general consideration, where we shall always employ reduced dimensionless quantities. But we shall keep this in mind in those concrete cases, where numerical values of parameters are important.

As said above, if the society consists of two groups only, cooperators 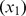 and defectors 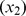, the latter always outperform the former, so that the sole evolutionary stable state is 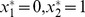. This unrealistic conclusion is caused by a tacit assumption that there exist unlimited resources supplied from somewhere outside.

Contrary to this, we consider a *self-organized* society, whose means of survival are those that are produced inside the society itself. A closed self-organized society cannot exist by just being composed of defectors because they will have no means for subsistence. To correct such an unrealistic conclusion, one could introduce punishers. But, from a self-organization view point, the introduction of punishers is not compulsory since, even without them, defectors cannot proliferate without bounds because of the lack of resources for their existence [56], [57].

It is necessary to note that the non-survival of cooperators can disappear, when one considers interactions on a network. Thus, computer simulations of cooperator games on lattices have demonstrated that cooperation can evolve in structural populations when cooperators interact more frequently with each other than with defectors, and share benefits of mutual cooperation [58], [59], or when individuals interact at different rates with each other by controlling the frequency distribution of the number of their partners [60], [61]. Cooperators survive on a network in the presence of modularity describing the separation of the population into clusters of different traits [62]. High modularity indicates a network with a strong clustered structure, where the interactions of individuals belonging to different clusters do not occur [63]. Modularity also arises, when the rate of interaction between different groups is not the same for all groups [63], [64]. Modularity favours cooperation by limiting the interactions of individuals to the members of the same community [62].

The principal difference between our article and these previous computer simulations is that we demonstrate analytically and parsimoniously the survival of cooperation without considering numerical calculations on networks. We show that the finiteness of resources is sufficient to limit the proliferation of defectors, without the need for heterogeneous interactions and clustering. While the latter ingredients are certainly relevant in many situations, our focus on the finiteness of resources is justified because it is often the first and dominant variable influencing population dynamics.

In the present paper, we combine some of the ideas used in the theory of biological populations, system theory, and utility theory and suggest a novel model of group evolution in a complex *self-organized* society, which strives to be more realistic than those usually studied in game theory. The main idea of our approach is in taking into account the bounded rationality of realistic agents [65], [66]. Accordingly, the utility of an agent is not an absolutely fixed notion, but is instead a relative characteristic defined with respect to the existing constraints. In our approach, instead of just one utility function for each group, we introduce relative utilities of a group with respect to the society as a whole as well as mutual utilities with respect to each other.

As an example, we consider a complex structured society composed of the trait groups representing four types of typical agents: *cooperators, defectors, regulators*, and *outsiders*. Concrete numerical analysis is given for the societies composed of two groups (*cooperators and defectors*) and of three groups (*cooperators, defectors*, and *regulators*). The role of outsiders will be studied in a separate publication.

## Evolution equations

### Holistic system-theory approach

As we have emphasized in the Introduction, we follow the ideas underpinning the theory of systems [1], [7]–[13], whose basic point is the holistic approach for describing systems. Accordingly, the primary unit is the system as a whole, as opposed to the reductionist approach, where a system is treated just as a collection of separate parts. In the holistic approach, a system is considered as a self-regulating organism, whose homeostasis is regulated by the system itself tending to reach and maintain a stable state. The regulation and control are achieved through positive and negative feedbacks. This approach is rather general, being applicable to different systems, physical, chemical, engineering, cybernetic, economic, social, and biological. In the present subsection, we delineate the main idea of the approach, leaving specifications to the following subsections.

Suppose that we aim at describing the behavior of a system consisting of several parts that, e.g., are characterized by fractions 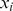, with 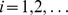. The fractions are the reduced quantities 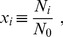
(2)whose specifications will be given below, and meanwhile their nature is not important, since the approach can be applied to various particular cases, as is mentioned above.

In the reductionsit approach, one characterises each fraction by its parameters and describes the system as a collection of equations containing only these parameters specific for separate fractions. An example of such an approach would apply to the collection of species, each characterised by its fitness and described by a replicator equation of type (1).

In the holistic system-theory approach, one starts with defining a *system functional* that characterises the system as a whole. Actually, this approach is standard and is known to be the most general and accurate in natural sciences, such as physics or chemistry, where a composite system is characterized by its energy, free energy, or Hamiltonian defining the system as a whole. In applied sciences involving control theory, a composite system is characterized by a cost functional. In economic, social, and financial sciences, it is more customary to characterize a system by the related utility. All these characteristics, whether *energy* 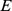, *cost functional* 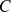, or *utility* 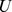 are functionals of the system parts, such as the fractions 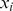 depending on time, 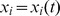. These characteristics are particular examples of the system functional. Generally, all of them are proportional to each other, thus, 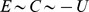. Therefore, it would be possible to deal with any of those system functionals. In what follows, keeping in mind biological and social systems, we prefer to work with the *system utility* that is a functional 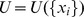. Since the variables 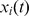 depend on time, this can be called a *dynamic utility functional* [67].

Equations, describing the behavior of the system parts, in the holistic system-theory approach, are derived by varying the system functional, such as energy or cost functional. Recall again that this is the standard way in natural sciences, where the evolution equations for system parts are obtained through the variation of a system functional, e.g., energy, Hamiltonian, or Lagrangian. Even the Heisenberg commutator equations can be shown [68] to be equivalent to variational equations for the total system Hamiltonian.

The system tends to its stable state that requires the increase of its utility. Hence, a fraction 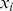 increases with time if its variation leads to the increasing system utility. This implies that 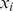 rises when the variational derivative 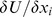 is positive, while 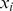 diminishes with time if 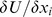 is negative. It is straightforward that this relation, taking into account possible external influxes 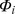, can be represented in the form 
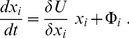
(3)


A self-regulating system self-organizes through its internal feedbacks, acting so that its utility would not diminish. Such feedbacks are defined by the linear responses 
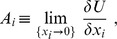
describing the direct influence of the fraction variations on the system utility. From this definition, it is clear that, when the increase of the fraction 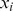 directly results in the increase of the system utility then, the feedback response 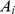 is positive. And if the rising 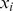 leads to the utility decrease, then the response 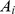 is negative. In addition to the direct influence on the system utility of the fraction variations, these fractions are correlated with each other through the correlation matrix, which is proportional to 
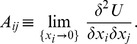



By construction, it is symmetric such that 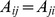.

From the above discussion, it immediately follows that the general form of the system utility reads as 
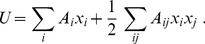
(4)


Again we recall that such a quadratic form is typical for the system energy in natural sciences or for the cost functional in control theory and engineering applications. It is also worth mentioning the Lande-Arnold [49]–[51] quadratic fitness for biological systems. With the system utility (4), we come to the evolution equations for the fractions: 
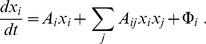
(5)


We stress that the signs of all parameters are uniquely defined by the conditions of whether the fraction variations increase or decrease the system utility. Thus, the response 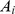 is positive if the increase of the fraction 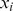 increases the system utility, while 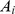 is negative when the fraction 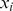 decreases this utility.

In this way, the parameter signs are not artificially chosen but are prescribed by the used holistic approach. Moreover, it would not be correct to say that the behavior of solutions is predetermined. Since, for instance, though the feedback of defectors on the society utility is negative (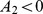), but the input of cooperators to the utility of defectors is positive (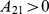). Therefore the actual behavior of the solution describing defectors is not at all predetermined, but depends on the delicate balance between the values of all system parameters.

### Utility rate equations

Let us now make concrete the general approach, which was described in the previous subsection, to the case of social and biological systems. We consider a society composed of the trait groups, or strategy groups, enumerated by an index 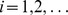, each consisting of 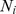 members. Each group will be characterized by the utility of the group with respect to the whole society and with respect to other groups. The concrete form of this dependence will be defined below.

In general, there can exist an influx 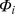 from outside to each group population. Such an influx can represent, for instance, pathogens or viruses infecting a biological organism. In the case of social systems, the influx can correspond to foreign invaders and terrorists.

The number of agents in a group can be very large, so that it is standard to consider the relative fractions 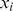 of the group populations normalized to a constant number 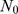, as in Eq. (2). If the total population of all groups would be fixed, it would be possible to interpret the normalization constant as the total population. However, in general, the group populations can grow or decay so that the total population is not necessarily fixed. In addition, there can exist external influxes of populations, not allowing for the conservation of the total population. Hence, fixing the total population would severely limit the admissible scenarios. However, it is always possible to choose for the normalization the amount of the total population at a given time. The most natural way is to accept for the normalization the total population 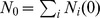 at the initial time 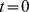, which therefore sets the scale for the population size.

The total population of any society is certainly finite. In that sense, the variable 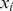 could be treated as discrete. However, when one considers large societies, as we keep in mind here, when the population can be of order of many thousands or millions, it is standard to treat 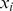 as a continuous variable.

In the biological literature, one usually characterizes the species by their fitness. And in the literature on social systems, one often employs the notion of utility. While the classical economic approach often defines the utility of an agent as the present value of future consumption, inter-generational extensions include bequest and the consumption of future generations. It is often argued that one of the most important measure of success is the passing of genes to future generation [69], [70], so that utility, as a measure of happiness, and fitness, as a measure of success, become entangled concepts. We prefer to use the term utility, keeping in mind applications to both biological as well as social systems.

The evolution equations (5) possess the form of the Lotka-Volterra rate equation. However, the basic novelty of our way is that we employ the system-theory holistic approach, as described above, which suggests a different interpretation of the coefficients that are defined not by some interactions between the group members, but by the utility of each group with respect to other groups and to the whole society. This prescription is important for correctly identifying the signs of the coefficients. Thus, the term 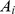 represents the utility of the 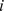-group to the society as a whole and the terms 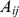 characterize the mutual utility that the 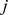-th group provides to the 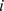-th group.

The evolution equations, we have just formulated, are the *utility rate equations*. For these equations, the number and signs of the coefficients are uniquely defined by the mutual utility of the groups, as is explained above.

It is worth emphasizing that the definition of the response 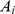 as being not purely intrinsic, but dependent on the relation of the 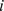-th population to the overall system, playing the role of environment for that population, is not merely admissible, but rather necessary.

The form of Eq. (5) is typical of many equations employed for describing the evolution of different biological species, where the role of the rate 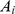 is played by the species fitness. As examples, we can mention the replicator equation (1), the Eigen [71], [72] and Crow-Kimura [73], [74] equations, and other models reviewed in Refs. [75]–[79].

The population variation rate, as well as fitness, are never completely intrinsic, but strongly depend on environment carrying capacity, that is, on the available resources, their quantity and quality [80]–[83], the existence of other coexisting species [84], and even on the predictability of environmental changes [85], [86]. The environment essentially influences the species fitness that can increase or decrease and sometimes unfavorable environments result in the fitness drop and species extinction [87].

Recall that the general definition of fitness, from the very beginning [88]–[90], has always been stressing its dependence on surrounding: “Fitness is the ability of an organism to survive and reproduce in the *environment*, in which it finds itself”. The dependence of the variation rate on surrounding is common for biological as well as social populations, for which the environment is given by the overall system, where the population exists [91], [92].

The rate can become purely intrinsic only for an imaginary species called Darwinian Demon, for which the rate is infinite [93]. But for any real population, the rate always essentially depends on the environment, where the population exists.

Moreover, the adjustment of each species to their surrounding by varying their fitness, i.e. the rate, is a basic pillar of the Darwin theory of natural selection, since all *organisms tend to exhibit considerable adaptation to their natural environments* [18], [94].

Thus, it is the generally accepted and the sole correct understanding that the population rate of any real species necessarily depends on environment, that is, on the system, where the population exists, including all properties of this environment. The population rate cannot be purely intrinsic, except the fantastic case of Darwinian Demon.

In our case, the role of the rates is played by the responses 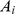 showing whether the given fraction increases or decreases the system utility, that is, the sign and value of each 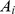 are unambiguously defined by the relative influence of the fraction on the utility of the system as a whole. This definition immediately follows form the system-theory holistic approach, as explained above.

### Specification of trait groups and utility relations

The above evolution equations can be applied to an arbitrary number of groups of different nature. In order to concretely demonstrate the validity of the approach, we consider a society formed by four groups: cooperators, defectors, regulators, and outsiders. These are defined as follows.

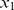
: *Cooperators*, who contribute to the society, working for the society benefit. In a human society, the cooperators form the actively working force producing the gross domestic product, because of which they can also be called producers. In a biological system, cooperators can be associated with healthy cells.

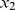
: *Defectors*, who, voluntarily or involuntarily, do not contribute to the society, and can exist only owing to the work of cooperators. In a social system, the groups that benefit from the society support without contributing are prisoners, pensioners, and unemployed people. In a biological organism, defectors can be represented by ill cells. Of course, there are many degrees interpolating between fully contributing and non-contributing behaviors and this classification is obviously made for the sake of simplification.

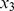
: *Regulators*, who maintain order in the society and punish defectors and harmful outsiders. In a human society, this role is played by the police, the army, and the order enforcing bureaucracy. To support the existence of regulators, the society and cooperators have to pay the necessary costs. In a biological organism, regulators can correspond to the cells of the immune system.

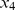
: *Outsiders*, who also exploit the society, as defectors, but with the difference that they enter the society from outside. These exploiting harmful outsiders should not be confused with immigrants. The latter, as soon as they become members of the society, separate into cooperators, defectors, and regulators. The harmful outsiders could be compared with those terrorists that are supported by external sources. Outsiders could also be interpreted as foreign raiding groups or even invading armies. For biological organisms, outsiders could be pathogens or viruses infecting the organism.

This classification uniquely determines the signs of the coefficients entering the evolution equations (5) as follows. We assume that the external influx can exist only for the outsiders and is positive (net in-flux). The diagonal terms 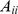 can describe mutual competition or cooperation. It is negative in the former case and positive in the latter case. In the presence of a dominant mutual cooperation, the positivity of the diagonal terms 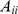 entails the unbounded increase of the population of the 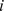-group [95], [96]. But, any real system has only finite resources and it is necessary to take into account competition mechanisms that will eventually dominate to limit population growth. This means that the diagonal coefficient 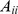, in general, could be described as a function of the population fraction 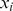, such that it would be positive for small 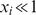 and changing its sign when 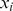 becomes larger. However, there is the simpler way to capture this phenomenon without introducing the sign change or higher-order terms with negative coefficients. This can be done by always considering the diagonal terms 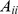 as being negative. Since these terms enter the equations being factorized with 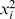, at small 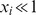, such terms 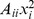 do not play an essential role, as compared to the linear terms 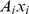. And at larger 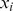, the former terms makes it impossible an infinite growth of populations.

In this way, keeping the diagonal terms negative provides mathematically the same effect as if they would change the sign. Moreover, the existence of competition between members of a group is a general phenomenon. This is natural even for cooperators, not talking about other groups. Really, when the number of cooperators would grow without limits, overpassing the available resources, they would certainly start competing with each other for subsistence. This does not contradict the fact that they do produce resources for the society. Real cooperators, or producers, are always of dual nature, from one side, cooperating in the process of production, but, from the other side, competing for the products.

The signs of all other coefficients are prescribed by the related utilities. Summarizing, we have the following signs.

Cooperators: 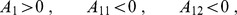


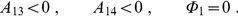
(6)


Cooperators are useful for the society, hence 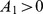. Defectors are not useful for cooperators, hence 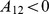. Regulators require the support of cooperators which is a cost to cooperators, which defines 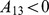. And harmful insiders also are not useful for cooperators, that is, 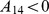.

Defectors: 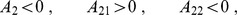


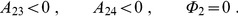
(7)


The meaning of the chosen signs is again straightforward. Defectors are not useful for the society, therefore 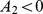. Cooperators are necessary for defectors who live at the expense of the former, hence 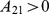. Regulators suppress and punish defectors, that is, 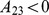. Invaders are not useful for defectors, so that 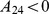.

Regulators: 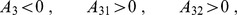


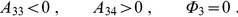
(8)


Society needs to support regulators at a cost, thus 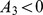. Cooperators, contributing to the society, are necessary for regulators, hence 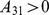. The role of regulators is to maintain order and to punish defectors, whose presence justifies the existence of regulators, resulting in 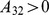. Similarly, regulators suppress harmful invaders, which justifies the existence of regulators, giving 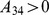.

Outsiders: 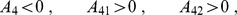


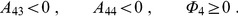
(9)


Harmful outsiders are not useful for the society, which means 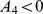. But cooperators are necessary for outsiders, hence 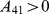. To some extent, outsiders exploit defectors by taking a part of their share, and benefit from their presence, hence 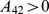. And, of course, regulators, suppressing outsiders, are not useful to them, which implies 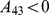. Only outsiders are here considered to contribute an influx 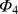 to the system.

Let us emphasize that all coefficients above are always treated as the corresponding utilities, or more precisely, as utility rates. And the variables 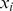 everywhere are the group population fractions defined in Eq. (2).

In specifying the relations between different groups, we should emphasize the important symmetry principle [14], [15], as a mechanism to ensure the structural stability of the dynamical system. According to this principle, each term containing 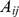, entering the system of equations, must have its counterpart containing 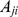. This principle preserves the action-counteraction symmetry that is responsible for the system structural stability. Note that the symmetry principle does not impose that the matrix 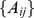 is symmetric, but just that if 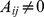, then so is 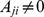. We are not aware of rigorous theorems proving the above claim, but we have observed its relevance by developing a large number of numerical simulations of multi-dimensional dynamical systems [14], [15]. In the construction of the dynamical systems presented here, we use this structural symmetry principle.

It is worth stressing that the signs of the coefficients are defined by the utility relations of groups with respect to each other and with respect to the whole society, but not by the interactions between the members of different groups. Though, in many cases, the signs of the interactions may be the same as those defined by utilities, but this is not always so. For instance, the interaction of cooperators with defectors could produce positive terms proportional to 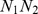 in the equation for regulators, implying that this interaction should lead to the increase of the numbers of regulators. Or, some defectors, and even outsiders, after dealing with regulators, could become cooperators, which would require to consider positive terms proportional to 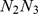 and 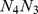 in the cooperator dynamics. It is possible to enumerate a variety of such admissible interactions. If we would treat the evolution equations as standard rate equations, whose coefficients are prescribed by interactions, this would make the description not merely overcomplicated, but also not well defined, because of the great number of all possible interactions that could be taken into account. In addition, such terms, describing various possible interactions, could break the action-counteraction symmetry of the system, making it structurally unstable. Similarly, the coefficient 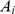 characterizes not the birth-death rate of the related population but the utility of the corresponding 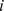-th group for the society. This is why 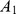 is positive because of the usefulness of cooperators for the society, while 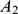 and 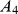 are negative, since defectors and harmful outsiders exploit the society. Regulators do not produce goods that would contribute to the society, but require costly support from it, hence 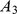 is negative.

The above classification is summarized by the following utility rate evolution equations, in which the signs of the coefficients are explicitly taken into account. As a result, we come to the following equations for the cooperators, 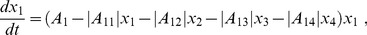
(10)for defectors, 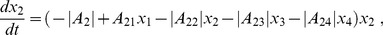
(11)for regulators 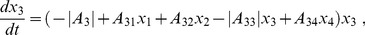
(12)and for the outsiders, 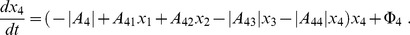
(13)


All coefficients not bracketed by an absolute value are positive.

### Definition of relative utilities

The dimensionless fractions, corresponding to trait groups, are defined as normalized with respect to the total number of all group agents 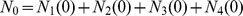 at the initial time 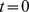. Therefore, the initial conditions for the group population fractions 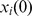 satisfy the equality 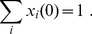
(14)


But the values 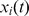, as functions of time 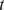, are not generally restricted to a simplex, i.e., their sum does not necessarily remain exactly equal to 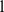.

A priori, Eqs. (10) to (13) look rather complicated, containing 20 unknown parameters representing relative utilities that need to be defined. Strictly speaking, it is possible to consider different situations, defining the parameters according to particular situations. Below, we suggest a general method leading to explicit relations between the parameters, thus essentially decreasing their number.

First, let us recall that the diagonal quantity 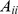 has been introduced in order to describe the competition between members of the same 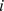-group. This is the competition for the available resources allocated for the particular group. On the other hand, the cost/benefit of an 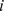-group to the society is given by 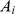. Hence, this group competes for the resources 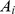 allocated to it. In other words, the amount of resources 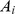 is proportional to the group carrying capacity. This implies that the diagonal term 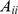, describing the group internal competition, should be taken as anti-correlated with, or more generally varying inversely to, the carrying capacity 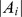. A convenient form is to take 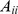 as being inversely proportional to 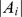, so that 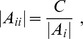
where 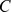 is a positive constant.

In a self-organized society, the available resources are those that are produced by cooperators, who are the sole net contributors to the society in term of resources and who compete for these resources. This is equivalent to saying that 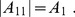
(15)


Then, comparing the above two equalities, we get the constant 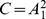. From here, we obtain the general relation for the diagonal terms 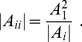
(16)


Nondiagonal terms 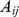 characterize mutual utilities of groups with respect to each other. There exist several expressions for mutual utilities [97], [98], among which the most symmetric one is the Bernoulli-Nash mutual utility 
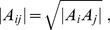
(17)which we adopt in what follows.

The Bernoulli-Nash mutual utility relates the mutual utility of different groups through their utility to the society to which they all belong. Different groups influence each other by influencing the society. For instance, if the 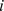-group is not useful to the society, so that 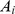 is zero, then this group is certainly not useful for the 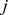-group, since the latter is part of the same society. The geometric mean for characterizing the mutual utility automatically ensures these properties and additionally conserves the dimensionality of all terms involved. Note that this form of the mutual utility, called the Bernoulli-Nash utility, is often used in various applications [97], [98].

Let us introduce the dimensionless parameters 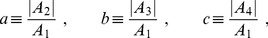
(18)quantifying the resources consumed by the corresponding groups, and the dimensionless outsider influx 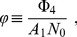
(19)all of which are non-negative quantities. And let us measure time in units of 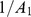. Then Eqs. (10) to (13) reduce to the system of equations 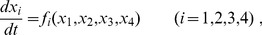
(20)with the right-hand sides 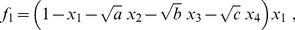
(21)

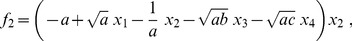
(22)

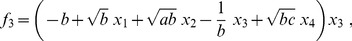
(23)

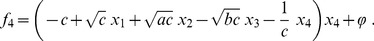
(24)


These equations are assumed to be complemented by the corresponding initial conditions 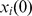. By definition, the fractions 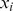 are non-negative. Therefore, we shall be looking only for non-negative solutions of the evolution equations (20) with (21–24).

## Evolutionally stable strategies

### Single-group evolution

Let us start the analysis with the case where, at the initial time, there is just one group and other group fractions are set to zero. Suppose, first, that there exists just the group of cooperators. It follows from Eq. (20) with (21) that the cooperator population is described by the logistic equation 
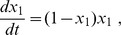
whose solution is 
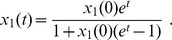



Substituting here the initial condition 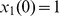 yields 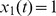 for all times 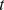. This is clear since the initial condition coincides with the stable fixed point 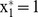. The meaning of this solution is that cooperators can perfectly exist without other groups.

Let us now consider the population of any other group, except cooperators, as existing alone at the initial time. For instance, let us take the equation for defectors, 
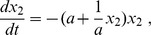
with the initial condition 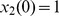. The solution is 
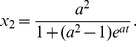



This population decays to zero with time. This is easy to understand as far as defectors do not produce but merely consume and cannot survive being left alone. The same result holds for other groups, i.e., for 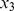 as well as for 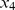, which cannot survive without cooperators.

### Coexistence of cooperators and defectors

More interesting is the case of two coexisting groups, cooperators and defectors. Actually, this is the classical situation from which one usually starts analyzing group interactions. In such a case, Eqs. (20) reduce to two equations 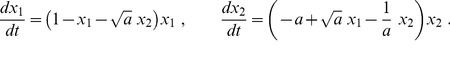
(25)


This system possesses two evolutionary stable states. One is given by the stationary solution 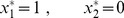
(26)that is stable when 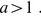
(27)


The meaning of this state is evident: if defectors attempt to consume more than cooperators can produce, the stable state can exist only when the very greedy defectors are absent, and only cooperators live.

Another evolutionary stable state is 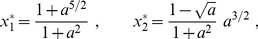
(28)being stable under the condition 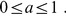
(29)


**Figure 1:**
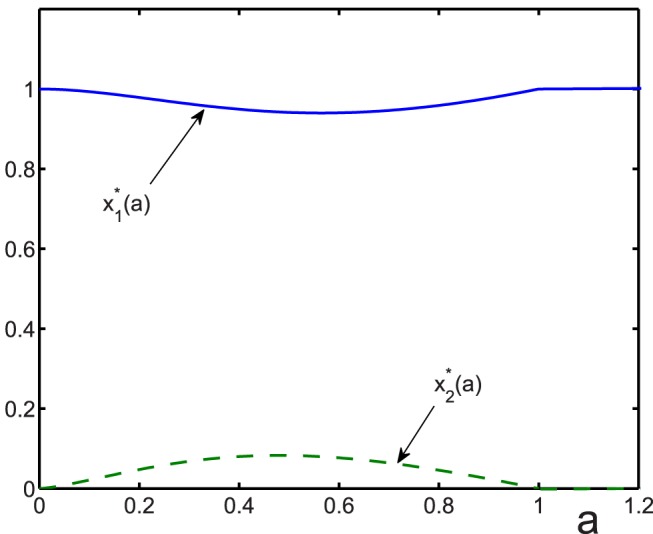
Evolutionary stable fractions of cooperators 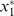 (solid line) and defectors 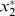 (dashed line) as functions of the relative amount of resources 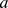 consumed by defectors, in the case of the coexistence of two trait groups, cooperators and defectors.

The meaning of this state is again rather clear. When defectors consume only a part of what is produced by cooperators, they can coexist with them. The dependence of the evolutionary stable population fractions 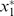 and 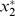, on the amount of the resources 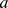 consumed by defectors, is shown in Fig. 1. The minimal fraction of cooperators happens when 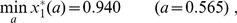
(30)while defectors have a maximum 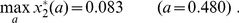
(31)


These minimum and maximum do not coincide. In a stable society consisting of cooperators and defectors, the fraction of defectors cannot overpass the limit of order 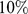.

These logical conclusions are drastically different from the result of the replicator equation for the case of two coexisting groups (cooperators and defectors), which predicts that the sole stable state consists solely of defectors, without any cooperators. Such a state is obviously impossible in a self-organized society since, without cooperators, nothing is produced and defectors would have no means for survival.

### Coexistence of three groups

Let us now consider a society formed by three trait groups, cooperators, defectors, and regulators. The evolution equations (20) form a three-dimensional dynamical system 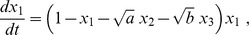


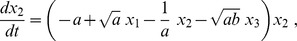


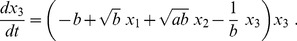
(32)


Looking for stationary solutions, we select positive and stable fixed points, employing the standard Lyapunov analysis. The system possesses four types of evolutionary stable solutions.

The first stable state is given by the fixed point 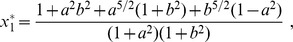


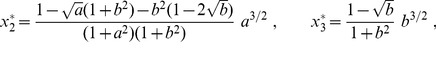
(33)which is stable under the conditions 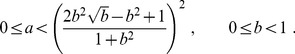
(34)


This stable state occurs when defectors and regulators consume reasonable amount of the resources of the society, so that all three groups can coexist.

The second stable state corresponds to the solution 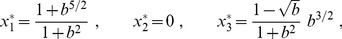
(35)being stable when 
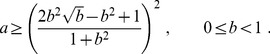
(36)


Here, the defectors, who consume too much die out, while cooperators and regulators coexist with each other.

The third stationary solution is 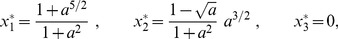
(37)which is stable if 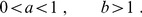
(38)


In this case, the excessive consumption (or cost) of regulators makes them unprofitable for the society, so that they are suppressed to zero, while defectors who are not too greedy remain viable at an optimal finite fraction 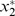.

Finally, the fourth state is described by the set 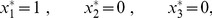
(39)which is stable provided that 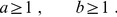
(40)


The meaning of this solution is again clear. Defectors and regulators, trying to consume more than what the cooperators produce constitute an unsustainable burden for the society, which prefers to eliminate them.

**Figure 2:**
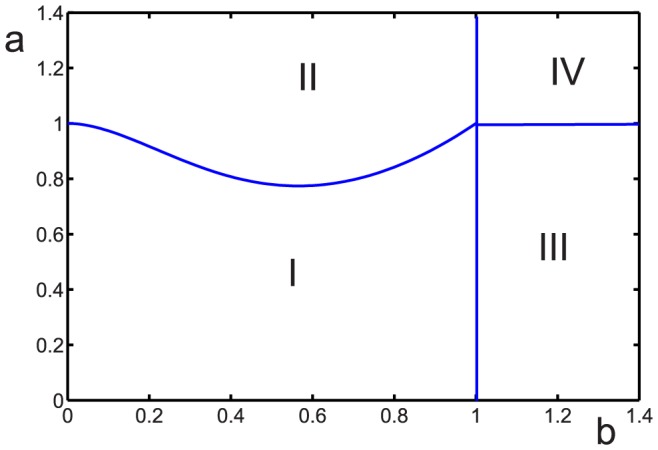
Phase portrait for the evolutionary stable states in the case of three coexisting trait groups, cooperators, defectors, and regulators on the plane of the relative amount of resources consumed by defectors 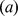 and regulators 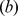. In region I, all three groups coexist, having nonzero fractions. In region II, there exist only cooperators and regulators, while there are no defectors 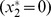. In region III, cooperators and defectors coexist, but there are no regulators 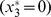. In region IV, only cooperators are present, without either defectors or regulators 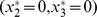.

**Figure 3:**
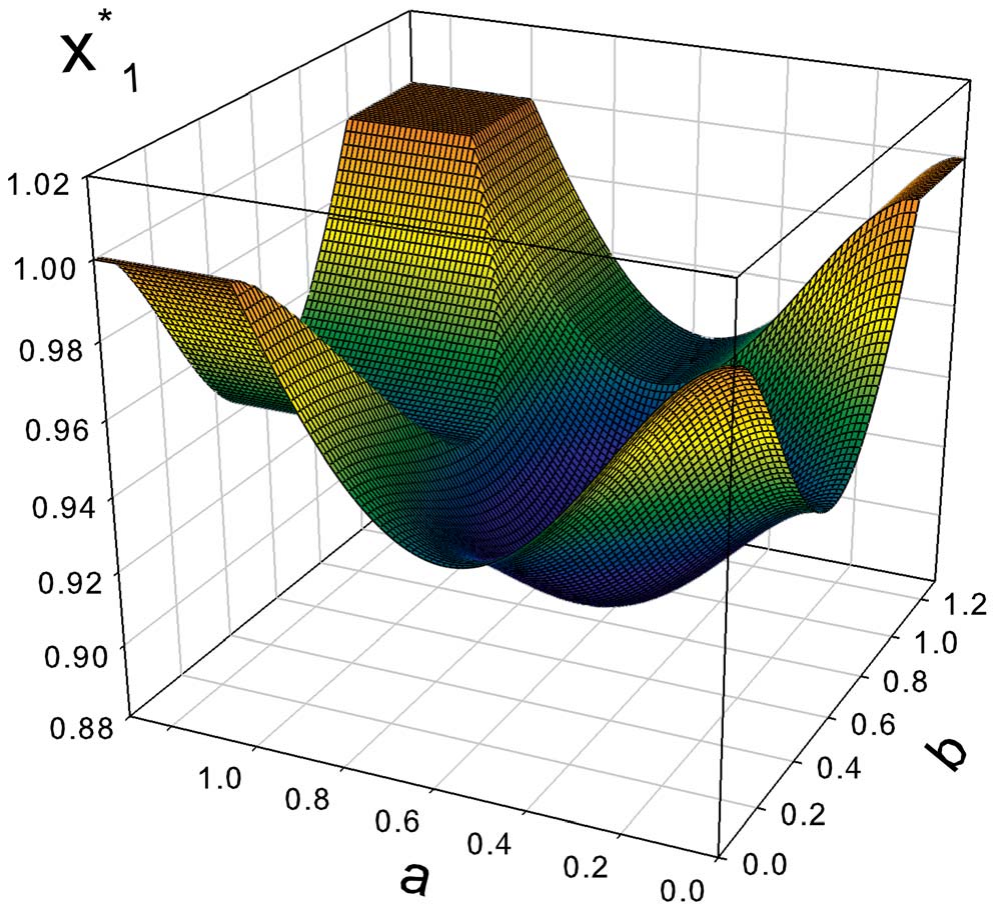
Evolutionary stable fraction of cooperators 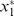 as a function of the parameters 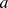 and 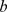.

The phase portrait for the case of three coexisting groups is shown in Fig. 2. Figure 3 shows the dependence of the evolutionary stable cooperator fraction 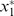 as a function of the amount of the resources consumed by defectors 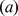 and regulators 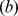. The function 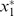 has a global minimum 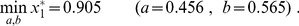
(41)


**Figure 4:**
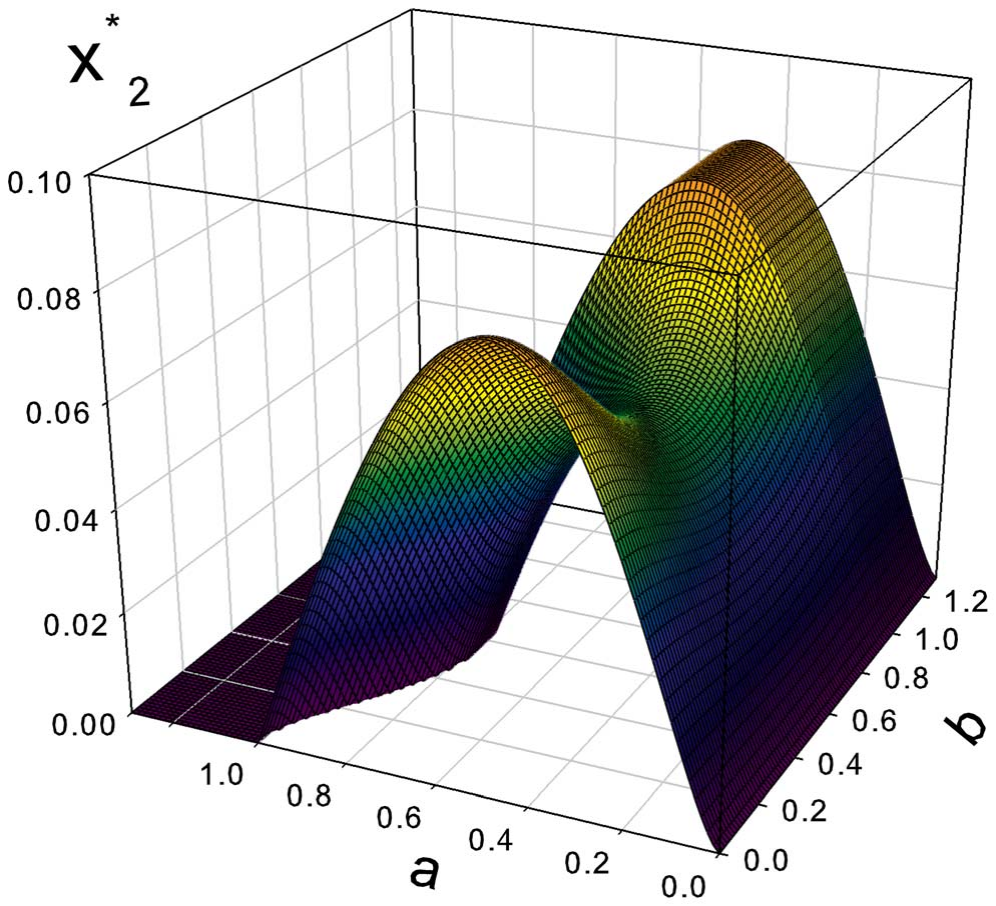
Evolutionary stable fraction of defectors 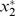 as a function of the parameters 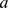 and 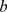.

**Figure 5:**
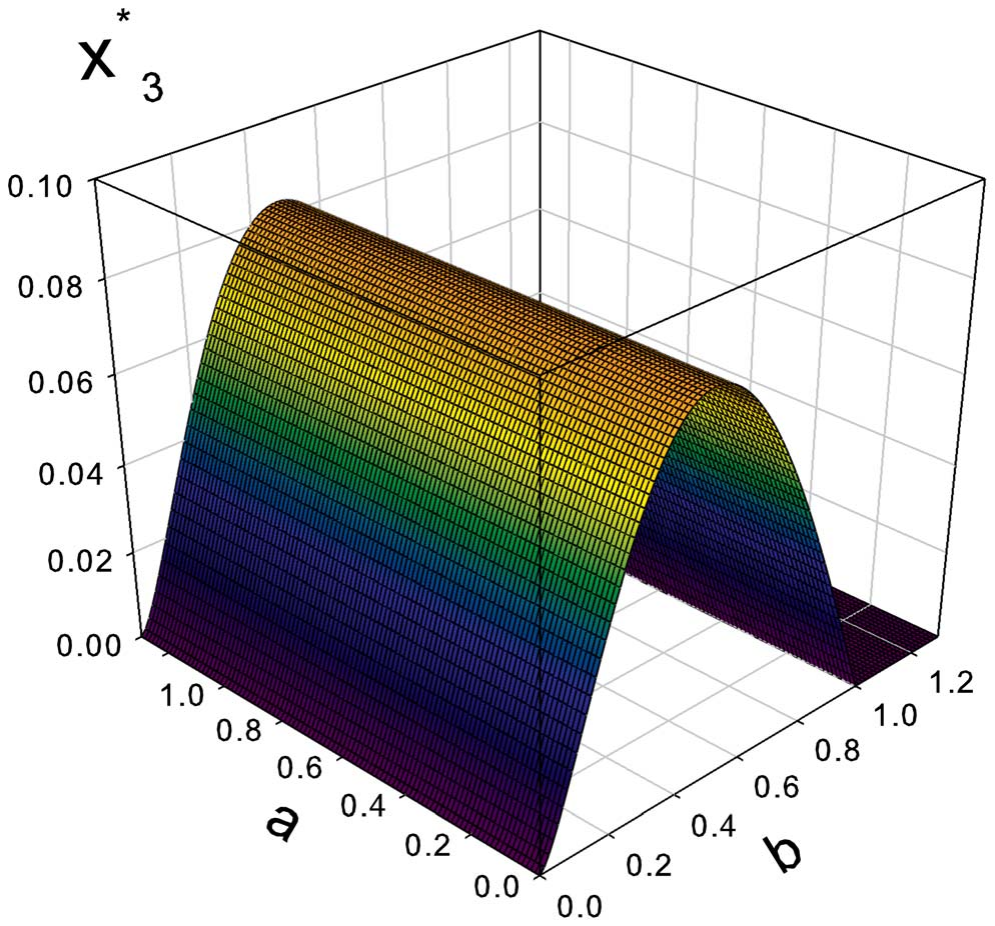
Evolutionary stable fraction of regulators 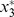 as a function of the parameters 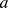 and 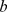.

The evolutionary stable fraction of defectors 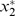 is shown in Fig. 4 as a function of 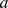 and 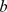. This function enjoys a global maximum 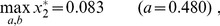
(42)when either 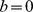 or 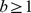. The stable fraction of regulators 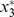 is presented in Fig. 5. This function has a global maximum 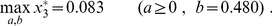
(43)


These results show in particular that, in order to achieve an evolutionary stable state in a self-organized society, the fractions of defectors and regulators should not exceed about 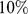 each.

## Applications to biological and human societies

### Biological societies without defectors

As follows from the above analysis, there can exist societies with no defectors. A good example of this is a bee colony. A honey bee colony typically consists of three kinds of adult bees: workers, drones, and a queen [99]. Several thousand worker bees cooperate in nest building, food collection, and brood rearing. Each member has a definite task to perform, related to its adult age. But surviving and reproducing take the combined efforts of the entire colony. Individual bees (workers, drones, and queens) cannot survive without the support of the colony. Even drones (males honey bees) are necessary members of the colony. In that sense, all bees are cooperators, having no defectors. They also do not have separate groups of regulators or defenders. Each bee perfectly knows its task. And in the case of danger, all bees defend their hive. But, excluding the cases of external aggression, in the normal situation, the bee colony is an example of a society where practically all members are cooperators. As an extreme, drones which are useful mainly in mating are expendable members of the colony, driven from the colony as winter approaches where they perish from cold and starvation. This mechanism removes defectors, in the sense defined in our model.

Another example of a biological society without defectors is a colony of ants. The colony is typically divided into the following castes, or classes: queens (reproductive females), males, and workers (nonreproductive females). Although there are great variations in social structure among ant colonies, the basic features are common to most species [100]. There are no defectors in an ant colony, all ants accomplish a job contributed to the whole group. Often, one classifies the workers into two castes, minor workers and major workers, or soldiers. However, this is done on the basis of the difference in their sizes, but does not mean that the sole job of soldiers is defending the nest, as they do accomplish other jobs [101]–[103]. All workers participate in the defense when necessary, though soldiers may do this more often. But soldiers also take part in other numerous acts, such as foraging food, taking care of larva, carrying dead nestmates, and so on [100], [104]–[106]. So, strictly speaking, soldiers are just bigger working ants. For this reason, they also are called foragers since, being bigger, they can carry heavier pieces of food.

In order to perform a quantitative comparison with our theory, we consider the ratio of the number of foragers (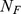) to the number of other working ants (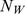), which is termed the cast ratio, 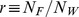. The fraction of foragers with respect to the total population is then 
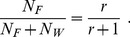
(44)


Defense is a part of the labor repertory, which can be denoted as 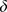. Then, the fraction of the defense trait reads as 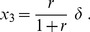
(45)


Recall that defenders are a part of the group of regulators.

The caste ratio of ants has been investigated in numerous articles (see the literature cited above and references therein). This ratio can vary in a wide range, depending on the colony age, time of the season, external danger, climate, and so on. To get estimates, it is reasonable to consider average numbers. Thus, the average caste ratio, measured for 503 colonies by Kaspari and Byrne [102], was found to be 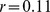, which gives the fraction of foragers 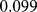. The repertory of ant labor has been intensively studied by Wilson [104], [105] and Oster and Wilson [100], who define the fraction of the defense behavior of foragers, among the total number of their acts, as 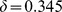. These data give the fraction of the defense trait (45) as 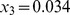. This value is nicely compatible with our estimate requiring that the maximal fraction of regulators in stable equilibrium should not be larger than 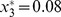.

One can thus consider an ant colony as a society with two trait groups, cooperators and regulators (soldiers). It seems that defectors rarely occur among social insects, such as bees, wasps, ants and termites. This is probably due to the strong selection pressure acting on these specific biological species, so that costly defectors are selected out. Defense is often necessary and one does find a non-zero defense trait group in an ant colony. But defense is in this case principally directed to external aggressions, in particular against other foreign ants for ant colonies, and occupies a rather small fraction of the population, as predicted by our model.

### Classification by distance from equilibrium

In real life, no society can be considered to be in an absolute stable equilibrium. Hence, we do not expect any society to lie exactly at or even close to any of the equilibrium points described above, either because of the previous history or because of external influences that may prevent the dynamics to have sufficient time to converge to the corresponding equilibrium point. How can we then characterize the relative stabilities of different societies and distinguish between them?

To answer this question, we propose to introduce the *distance from equilibrium*

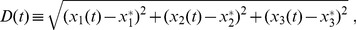
(46)and use this metric to classify societies in terms of their relative stability, according to whether they are further or closer to an equilibrium state.

### Application to human populations

In order to show that the proposed approach yields not merely qualitatively reasonable results, but also provides a realistic quantitative picture, let us consider the social systems represented by populations of different countries. Then, the fraction of cooperators 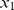 describes the active working part of the society, producing all resources. As defectors (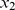), we count the fraction that does not take a direct part in the production of goods, but is supported by the society; these are unemployed people, pensioners, and prisoners. Regulators (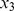) are police, army, and law-enforcing bureaucrats.

To define the parameters 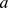 and 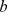, we resort to the analogy between human and biological societies. These parameters are defined in Eq. (18) through the quantities 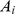 that symbolize the utilities of the related groups. Recall that utility for a human society is equivalent to fitness for a biological society. In biology, fitness is defined as the product of viability and fecundity rate [52]–[55]. Viability is the capacity for survival of a group [55], while the fecundity rate or potential reproductive capacity rate [52] is the number of offsprings produced by an organism per unit time.

Similarly, we define the utility 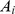 as the product 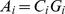 of the capacity 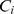 available to a group for its survival and of the growth rate 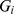 characterizing the relative group growth or its potential growth due to the increasing capacity. In human societies, the growth of a group is usually proportional to the increase of the resources provided to this group [107]–[110]. Conversely, increasing the group resources provides means for the potential group growth. In this way, the parameters 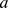 and 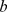 are defined as the ratios 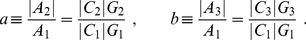
(47)


In more or less stable countries, the growth rate of the total resources produced by cooperators is assumed to coincide with the growth rates of the resources available for each group. Then, the system parameters become 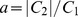 and 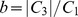. This is probably not always the case, and some groups can grow faster than others. An example could be the bureaucracy in Russia, which grows much faster than the country GDP. Another example may be the accelerated growth of the weight in percentage of GDP of government spending in western countries, including the US, as a reaction to the great recession that started in 2008. In many countries, the growth of spending programs, such as in Japan in the mid-1990s, or in health care, also grow faster than GDP. However here, for simplicity, we assume that the growth rates of the different groups are proportional to GDP, leaving the fine structure related to the possible unbalance between the growth rates for future economical investigations.

Within this assumption leading to 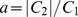 and 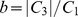, the parameter 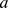 characterizes the portion of the resources, that is of the budget, the society pays for supporting the defectors, while 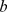 is the portion of the budget spent for the regulators. All these data can be found on the websites provided at the end of the list of References.

Note that different Internet sources, even the official ones, quite often give different information on a country total population, labor force, employment, number of military or police forces, and so on. We have used the data for 2008, since only those data were presented in the most complete and uniform way for the chosen countries. In those cases where we could not find the appropriate data for 2008, we used the information for the nearest available year. It was possible to do so because there were no sharp changes in numbers for adjacent years. The differences in data should provide only small deviations in initial conditions or parameters and, because of the structural stability of the system (32), it should not drastically change the results. For example, to estimate the number of retired people in France, we used the data from the population pyramid of France for 2010, instead for 2008, to be able to take into account early retirements.

We do not expect any country to lie exactly at or even close to any of the equilibrium points described above, because there always exist changes of conditions at the geopolitical and economic levels that may prevent the dynamics to have sufficient time to converge to the corresponding equilibrium point. We use the distance from equilibrium (46) as the metric to classify the societies in terms of their relative stability, according to whether they are further or closer to an equilibrium state.

The initial values and parameters entering the system of equations (32) are taken from the data characterizing the corresponding countries. All these data can be found on the websites [111], [112]. For example, we illustrate below the case of Israel [111], explaining how the data, characterizing the considered countries, have been estimated.

The total population of Israel in 2008 is estimated as 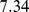 M. According to our definition, defectors are assumed to constitute the part of the population that does not contribute to the society, but instead is supported by it. These are pensioners, unemployed, and prisoners. Pensioners compose 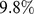 of the total population.

The labor force of Israel in 2008 is estimated as 2.96 M people. Unemployment rate is 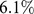 of its labor force, which equals 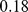 M of the total population of Israel. Hence, the percentage of unemployed with respect to the total population is 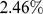.

The number of prisoners in Israel in 2008 is 0.013 M, which gives 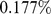 of the total population. Thus the percentage of defectors is evaluated, in total, as 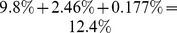, which gives 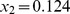.

The group of regulators is formed by the army, police, and bureaucrats. The army is strong of 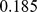 M persons and the police has a force of 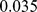 M people. This gives the total number of the population in the army and police as 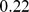 M. In other terms, the army and police compose 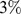 of the total population.

The number of bureaucrats makes 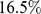 of the labour force of 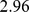 M. This gives the total number of bureaucrats in the country as 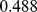 M. So, the percentage of bureaucrats with respect to the total population is 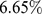. Therefore the fraction of regulators 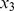 is evaluated as 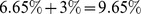, which gives 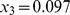.

The fraction of *cooperators*, 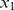, at the given initial time, is calculated as 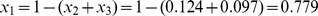. Note that this is different from the employed productive fraction, as we include all the children, who arguably grow human capital, and people working at home (housewives for instance) who do not appear in the unemployed statistics but nevertheless contribute to the production of the country.

The parameter 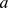 is the cost of the group 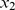 for the group 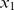, and the parameter 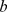 is the cost of the group 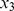 for the group 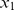. We consider 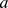 as the part of the budget spent on *social protection*. In 2008, Israel spent on social protection 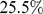 of the budget, which means that 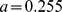.

On *national defense, public order and safety*, and *state governing*, Israel spent respectively 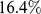, 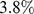, and 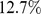 of its total budget. In total, it thus gives 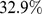, hence 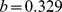.

In this way, analyzing the system of equation (32) for Israel, we employ the found parameter values 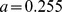 and 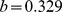, and take as initial conditions 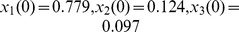.

We have followed the same procedure for the other nine countries. All data are taken from the websites [112].

As shown in the Table 1, we find for the ten analyzed countries that they all lie in the domain I of the phase portrait depicted in Fig. 2, for which there is a stable fixed point with a finite fraction of all three groups of cooperators, defectors and regulators. This is not surprising, since the common structure of all countries always includes these three groups. We are not aware of countries that would not contain some of these groups. This is in contrast to social insect colonies, for which defectors are too costly and rarely exist, as discussed for the cases of bee and ant colonies.

**Table 1:** Data for different countries corresponding to the present fractions of cooperators 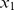, defectors 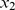, and regulators 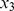, compared to their stable stationary solutions (given in brackets).

	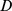	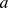	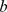	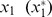	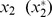	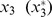
1. Israel	0.173	0.255	0.329	0.779 (0.933)	0.124 (0.050)	0.097 (0.073)
2. Japan	0.203	0.350	0.192	0.761 (0.940)	0.157 (0.068)	0.082 (0.046)
3. Switzerland	0.217	0.407	0.197	0.750 (0.934)	0.186 (0.071)	0.064 (0.047)
4. USA	0.242	0.190	0.304	0.740 (0.945)	0.167 (0.039)	0.082 (0.069)
5. Germany	0.282	0.451	0.196	0.704 (0.919)	0.240 (0.072)	0.056 (0.047)
6. France	0.284	0.414	0.193	0.690 (0.934)	0.205 (0.072)	0.105 (0.046)
7. Italy	0.286	0.385	0.250	0.697 (0.929)	0.233 (0.067)	0.070 (0.059)
8. Spain	0.304	0.340	0.187	0.691 (0.942)	0.238 (0.067)	0.071 (0.044)
9. Greece	0.314	0.365	0.294	0.665 (0.926)	0.234 (0.063)	0.101 (0.067)
10. Russia	0.471	0.363	0.225	0.546 (0.934)	0.322 (0.067)	0.132 (0.053)

The portion of the budget, consumed by defectors is 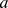 and the relative amount spent for regulators is 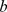. Countries are classified according to their distance 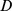 from equilibrium defined by expression (46).

The real value of the metric (46) rests in the possibility to quantify how far from their corresponding equilibrium point is each country, given its corresponding loss 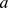 due to defectors and cost 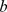 of regulators. In order to understand the meaning of the Table 1, one could interpret the coefficients 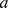 and 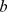 for each country as reflecting the choice of the society, for instance through evolution and/or a political process. In other words, we interpret the parameters 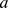 and 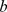 measured for each country as the independent variables determined by the society. Then, given the set 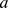 and 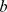, our theory predicts what should be the optimal fraction of the three groups of cooperators, defectors and regulators, where optimality is referring to maximum stability. Another possible interpretation is that the distance 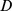 defined by (46) for each country quantifies a prediction of a future probable evolution of the three groups, if the relative losses 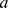 associated with defectors and costs 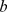 of regulators remain unchanged. Indeed, being at a distance to the stable fixed point, a given country is expected to see its population partition in the three groups to evolve towards the fractions given by the values of the stable fixed points for the measured 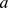 and 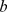. We are, of course aware that this may not happen, since the parameters 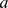 and 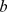 can be varied because of political reasons and technology changes.

Let us mention that it could be possible to consider the deviations from the stationary states for each of the variables 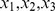 separately. This would show the relative instability of the country with respect to the particular characteristics. However, the overall stability is connected with all three deviations, since it is possible to compensate one large deviation by other deviations. Although the consideration of partial separate deviations is admissible, here we limit ourselves to the analysis of the global deviation 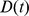.

Some interesting features are worth noting. For all countries, there are not sufficiently many cooperators, too many defectors and too many regulators, compared to the normative values associated with the stable equilibrium points. This suggests that societies choose to allocate their resources in a way that is suboptimal with respect to stability, considering other factors such as social preferences as well as historical culture, a hardly surprising observation. As a consequence, the possibility for changes and instabilities in the presence of external perturbations should not be underestimated, since it results from the fundamental choice taken consciously or through the force of history to function far from the stability point.

It may be surprising to find Israel at the top of the ranking. One could advance the interpretation that its special geopolitical situation has forced it to evolve closer to a regime of structural stability. Other countries in the West may have, at least for a while, the luxury to choose less stable policies.

Another interesting point is made by comparing Switzerland with USA and Spain. While the cost of defectors (main social and retirement costs) is much higher in Switzerland than in the USA (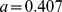 compared with 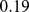), Switzerland is ranked higher (more stable) than the USA because it has a much lower cost for regulators (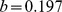 compared with 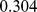 reflecting its much smaller relative military budget), confirmed by its smaller fraction of regulators. In contrast, Spain has lower costs from defectors and for regulators than Switzerland, but more defectors and almost twice as many regulators, hence its relatively much lower rank.

The laggard of the list is Russia. Examining its parameters 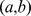 and its three group fractions 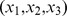, it is clear that its woes are rooted in the combination of having the smallest number of cooperators and the largest number of defectors, as well as the largest number of regulators. This points clearly to the general directions of reforms that could improve its state.

Finally, it is quite remarkable that the classification of countries presented in the Table, in terms of their distance from equilibrium, is in excellent agreement with the general understanding about their respective economic stability. It is particularly interesting that, for the European countries, the ranking shown in the Table coincides with the ranking in terms of GDP per capita given by the International Monetary Fund, which can be found on the site [113].

### Dynamics in presence of noise

Real societies can never reach their absolute equilibrium states because of the everlasting existence of external perturbations, which can be modeled as a kind of noise superimposed on the deterministic dynamics that we have considered until now. To investigate the consequence of such a noise, we have studied the behavior of the population fractions as functions of time in the presence of random perturbations that are modeled by adding to the evolution equations (20) Gaussian white noise characterized by the standard deviation 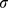. Then, each population fraction obeys the equation 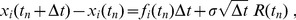
(48)where 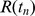 are random numbers, 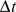, a small time interval, 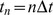, and 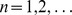.

**Figure 6:**
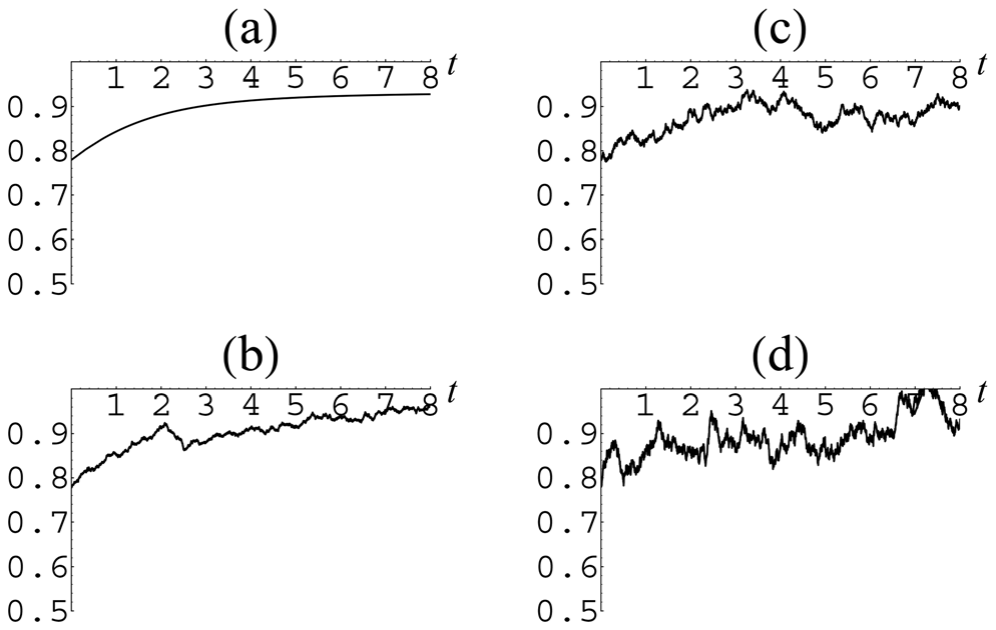
Temporal behavior of the cooperator fraction 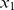 in the presence of noise of different intensity for the parameters 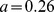, 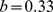 corresponding to Israel: (a) 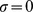; (b) 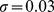; (c) 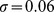; (d) 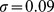.

**Figure 7:**
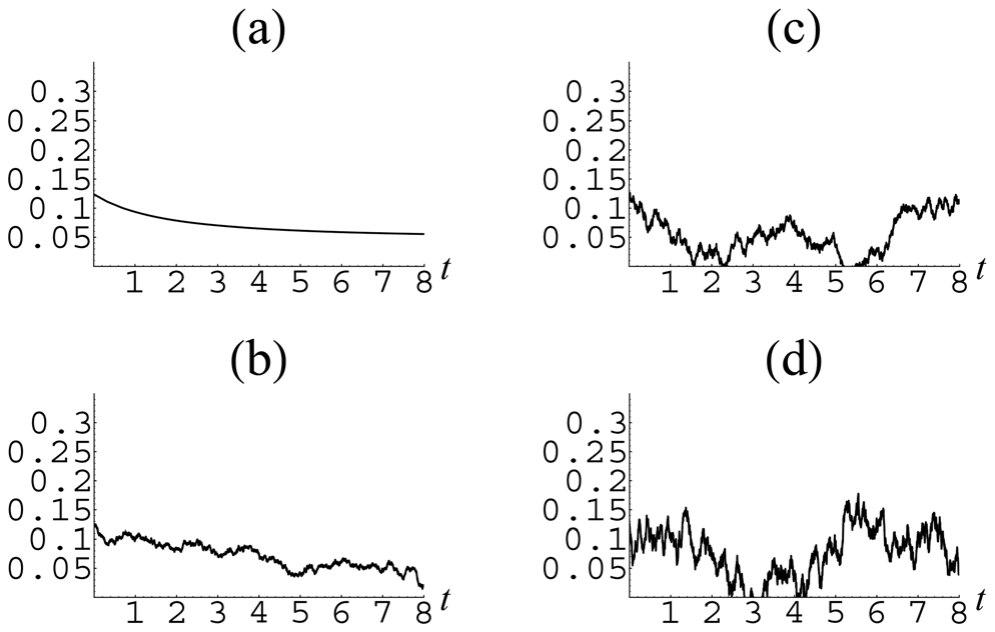
Temporal behavior of the defector fraction 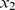 in the presence of noise of different intensity for the parameters 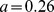, 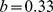 corresponding to Israel: (a) 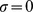; (b) 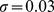; (c) 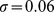; (d) 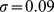.

**Figure 8:**
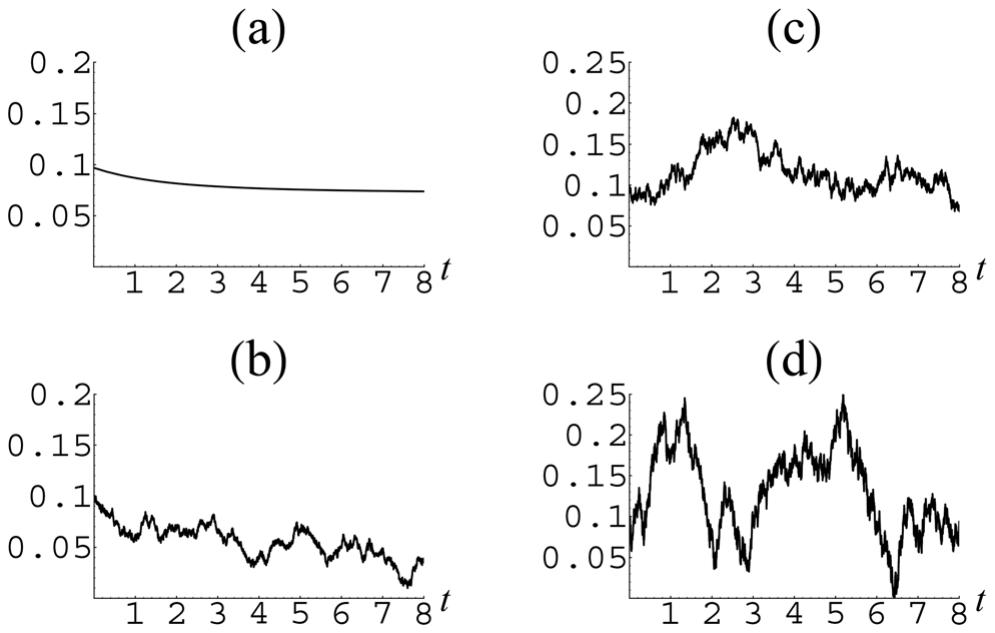
Temporal behavior of the regulator fraction 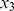 in the presence of noise of different intensity for the parameters 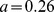, 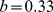 corresponding to Israel: (a) 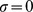; (b) 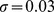; (c) 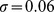; (d) 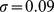.

For illustration, we take the data corresponding to Israel. The resulting temporal behavior of group fractions is shown in Figs. 6, 7 and 8. From these figures, it is seen that, in the presence of external perturbations, even a rather stable country, such as Israel, from time to time may experience quite strong deviations far from equilibrium.

## Conclusion

A novel approach has been suggested for describing the evolution of *self-organized* structured societies composed of several trait groups. The main idea is to consider the utility rate equations, whose parameters are characterized by the utility of each group with respect to the society as a whole and by the mutual utilities of groups with respect to each other.

The principal point is that we consider *self-organized* societies whose resources are those produced by the societies themselves.

We have analyzed in detail the cases of two coexisting groups (cooperators and defectors) and of three groups (cooperators, defectors, and regulators). In a self-organized society, neither defectors nor regulators can overpass the maximal fractions of order 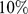. This is in contrast with the standard replicator equation, where defectors can overpass the amount of cooperators, if no regulators or punishers are present.

According to the studied equations, there can exist societies without defectors. Examples of such societies are rather common for biological species. For instance, there are no defectors in bee or ant colonies.

It is numerically demonstrated that the suggested approach, though being relatively simple, gives reasonable results when applied to ten countries, from Israel to Russia. We have shown in particular how the ranking of countries in terms of the distance to their corresponding stable equilibrium point is in remarkable concordance with more standard economic ranking based, for instance, on GDP per capita.

A possible generalization of the evolution equations can be done by considering the society behavior during long time periods, when the system parameters 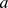 and 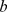 become functions of time. For example, the amount of the resources produced by cooperators in the long run can be taken as a function of time, similarly to the time dependence of the carrying capacity studied in other models [95], [114], [115]. In contrast, as has been explained in the Introduction, we have considered here the intermediate time scales lying between the short times of the interactions among the group members and the large scale of the system lifetime.

In the present paper, we have limited our considerations to the case of closed societies, when there are no invaders from outside. For social systems, such invaders could be associated with terrorists or foreign armies, invading the assembly of cooperators, defectors, and regulators. For biological systems, these invaders could correspond to viruses and pathogens penetrating a biological organism formed of healthy cells (cooperators), ill cells (defectors), and the cells of immune system (regulators). The role of such invaders will be studied in a separate publication.
